# Creation of the First Comparative Gluten Allergenicity Map Using a Mouse Model: A Preclinical Tool to Establish Substantial Equivalence of Novel Wheat Glutens

**DOI:** 10.3390/ijms27093716

**Published:** 2026-04-22

**Authors:** Rick Jorgensen, Haoran Gao, Harini Gangur Acharya, Maya Blanka Srkalovic, Chris Van Antwerp, Perry K. W. Ng, Venu Gangur

**Affiliations:** 1 Food Allergy and Immunology Laboratory, Department of Food Science and Human Nutrition, Michigan State University, East Lansing, MI 48823, USA; jorgen70@msu.edu (R.J.); gaohaora@msu.edu (H.G.); gangurhar@gmail.com (H.G.A.); msrkalovic6397@gmail.com (M.B.S.); vanant29@msu.edu (C.V.A.); 2 Cereal Science Laboratory, Department of Food Science and Human Nutrition, Michigan State University, East Lansing, MI 48823, USA; ngp@msu.edu

**Keywords:** gluten, wheat, allergy, anaphylaxis, comparative allergenicity map, mouse model, IgE, mast cell, hypothermic shock response, wheat genome

## Abstract

Gluten allergy is linked to high risk of anaphylaxis. The relative allergenicity of glutens (alcohol-soluble gliadin and acid-soluble glutenin) from the three commercially grown wheat species (diploid *Triticum monococcum*, tetraploid *Triticum durum*, hexaploid *Triticum aestivum*) is unknown. A comparative gluten allergenicity map (CGAM) from these species will enable the identification of potentially hyper-/hypo-/iso-allergenic species/varieties of wheat as well as the determination of substantial equivalence of genetically engineered (GE) or other novel wheat lines. Here, using a recently described novel mouse model, we tested the hypothesis that the three different wheat species will exhibit natural variation in their gluten allergenicity. Groups of Balb/c mice were transdermally sensitized to alcohol-soluble or acid-soluble gluten extracts followed by elicitation of systemic anaphylaxis. Initial studies were performed to validate the model for glutens from the three wheat species. Both glutens from all three wheat species elicited robust specific IgE responses, as well as systemic anaphylaxis. However, comparative mapping analysis revealed differences in capacity to elicit specific IgE among the three wheat species with *T. aestivum* being the most potent in both gluten extracts. Hypothermic shock response analysis revealed that the three species elicited similar kinetics and intensity of anaphylaxis. Nevertheless, when analyzing mucosal mast cell response, it was revealed that the glutens from *T. aestivum* emerged as the most potent elicitor. Collectively, these results yield the first CGAM that may be utilized for preclinical testing of the allergenic potential of glutens from novel (e.g., GE) wheats and processed wheat products against existing wheat glutens.

## 1. Introduction

The recognition of wheat as a common trigger for immune-mediated food allergies has become a significant global health concern, impacting both public health and food safety. Given the potential for life-threatening anaphylaxis, this presents a substantial health and financial burden [1]. A recent worldwide meta-analysis on wheat allergy revealed that 0.22% to 1.93% of individuals are affected by this condition [2]. The prevalence varies across different regions, relying on self-reported physician-diagnosed wheat allergy [2]. With no current cure for wheat allergy, individuals must adhere to strict elimination diets, leading to a compromised quality of life due to an increased risk of severe allergic reactions at any given time [3].

Gluten constitutes a group of proteins present in cereal grains like wheat, barley, and rye. Traditionally, these glutens are categorized based on their solubility properties into two main groups: ethanol-soluble prolamin proteins (gliadins, comprising 30–40% of total proteins) and weak acid-soluble proteins (glutenins, making up 45–50% of total proteins); the remaining soluble proteins in aqueous solutions are non-gluten proteins, specifically albumins and globulins [4,5]. Gliadins function as individual proteins interacting through hydrogen bonds, predominantly featuring intramolecular disulfide bonds. In contrast, glutenins are polymeric proteins forming connections through both intermolecular and intramolecular disulfide bonds. Additionally, gliadins can be linked to the glutenin network through intermolecular disulfide bonds [6]. Among the ethanol-soluble proteins, ω–1, 2, 5 gliadin, and α/β/γ-gliadins have undergone extensive characterization and are recognized for inducing allergic reactions in susceptible individuals [1,7]. Within the acid-soluble fraction, both high-molecular-weight and low-molecular-weight glutenin subunits can bind IgE antibodies causing severe allergic reactions [1,7,8].

There is substantial evidence indicating that wheat gluten can trigger various immune-mediated diseases, including gluten hypersensitivity (or allergy), celiac disease (CD), and non-celiac gluten sensitivity (NCGS) [4]. Among these, gluten hypersensitivity, also known as gluten food allergy or wheat food allergy, poses a threat to life [1,9,10]. Inappropriate activation of the immune system by wheat proteins, encompassing both gluten and non-gluten proteins, is responsible for hypersensitivity reactions to wheat [1,11]. The immune mechanisms and clinical manifestations of gluten hypersensitivity differ significantly from those of CD and NCGS. Gluten hypersensitivity is primarily attributed to the production of IgE antibodies against gluten during initial exposures, leading to their binding to and sensitizing mast cells and basophils. Subsequent encounters with the gluten cause their degranulation, resulting in potentially life-threatening anaphylaxis [6,12]. In contrast, gluten-induced CD is an autoimmune chronic inflammatory disease that predominantly affects the small intestine; in some instances, gluten can also trigger CD associated with dermatitis (referred to as dermatitis herpetiformis) and brain dysfunction (referred to as “hyper-excitable celiac brain”) [13,14,15]. NCGS manifests clinically as a chronic digestive disorder with unknown mechanisms, although activation of the innate immune system is implicated [16].

Genetically, modern wheats trace their origins to three genomes—A, B, and D [7]. The commonly consumed wheats fall into three wheat categories: diploid (AA genome; for instance, einkorn, and species *Triticum monococcum*), tetraploid (AABB genome, such as durum, and species *Triticum durum*), or hexaploid (AABBDD genome; exemplified by bread wheat and species *Triticum aestivum*) [7,17,18]. Hexaploid and tetraploid wheat encompass numerous species, each hosting hundreds and thousands of varieties and accessions [6]. Given that allergenic protein content and structure are genetically determined, variations in wheat allergenicity can be anticipated at the ploidy level, species level, and even at the variety/accession levels [19,20]. Currently, it is assumed that gluten from all wheat species have equal allergenic potential; however, there is no data to justify this assumption. This was the rationale for undertaking this study.

There is extensive evidence in the literature that industrial and food processing of glutens can alter their allergenicity [6]. A standardized comparative map of intrinsic allergenicity of native glutens from various species will be valuable to evaluate the impact of processing methods on gluten allergenicity. Such a map is unavailable at present—this was the focus of the present study.

Currently, genetically modified wheat, also known as genetically engineered (GE) wheat, is not commercially available. However, efforts are underway to develop these and get approval from regulatory bodies such as the United States Food and Drug Administration (US FDA) [21]. A major concern regarding the safety of such GE wheats would be clear elucidation of the allergenicity potential of glutens from such wheats. In aiding the allergenicity assessment of GE foods, international regulatory and health agencies, such as the Food and Agriculture Organization (FAO)/World Health Organization (WHO), have introduced the ‘substantial equivalence’ concept as a general guideline [22,23]. This concept serves as a tool to evaluate the allergenicity of GE foods, including GE wheats, by comparing them with their conventional non-GE native counterparts [23,24,25]. Although the FAO/WHO recommends the use of animal models in assessing in vivo allergenicity of GE foods, there is currently no validated comparative model available for testing allergenic potential of glutens from GE wheat [22].

The literature describes various animal models for gluten hypersensitivity, encompassing dogs, rats, guinea pigs, and mice [6,26,27,28,29,30,31,32,33,34]. In general, these models can be classified into two types: (i) adjuvant-based models and (ii) adjuvant-free models. Adjuvant-based models have been more prominent because historically, they were the first models to be developed, and they provide robust readouts of immune and allergenicity markers. However, they do not have the power to decipher inherent allergenicity potential of glutens due to enhancement of the immune response by the adjuvants that are co-administered with gluten. In contrast, the recently reported adjuvant-free models provide a unique opportunity to evaluate the intrinsic allergenicity potential of glutens from various wheat species [6,33,34].

In this study, we tested the hypothesis that the three different commonly cultivated wheat species (*T. monococcum*, AA genome; *T. aestivum*, AABBDD genome, and *T. durum*, AABB genome) will exhibit natural variations in their gluten allergenicity. The objectives of this study were as follows: (i) to validate the transdermal sensitization and systemic elicitation of the disease model using alcohol-soluble gluten extract from *T. monococcum* and *T. aestivum*; (ii) to develop comparative sensitization and disease elicitation maps of alcohol-soluble gluten extracts from the three wheat species, *T. durum*, *T. monococcum* and *T. aestivum*; (iii) to validate the transdermal sensitization and systemic elicitation of the disease model using acid-soluble gluten extract from *T. monococcum* and *T. durum*; and (iv) to develop comparative sensitization and disease elicitation maps of acid-soluble gluten extracts from these three wheat species.

Collectively, these results yield a comparative map of the natural variation in intrinsic sensitization and disease elicitation potencies of both types of glutens from three commonly consumed wheat species for the first time. This comparative map may be utilized for the assessment of the allergenic potential of glutens from novel wheats, such as GE wheats, and differently processed wheat products.

## 2. Results

### 2.1. Validation of the Transdermal Sensitization and Systemic Elicitation of Disease Mouse Model for Triticum monococcum Using Alcohol-Soluble Gluten Extract

#### 2.1.1. Transdermal Exposure to Alcohol-Soluble Gluten Extract from *T. monococcum* Elicits Robust Specific IgE Antibody Responses in Balb/c Mice

Groups of Balb/c female mice were allocated and exposed to alcohol-soluble gluten extract from *T. monococcum* (genome AA) or vehicle via transdermal application, following a repeated weekly exposure regimen as per Section 4. Blood samples collected before the first exposure (pre) and after the sixth exposure (6R) were subjected to analysis for specific (s) IgE levels. Notably, a significant rise in sIgE antibody levels was observed following transdermal exposure to alcohol-soluble gluten extract from *T. monococcum* in allergic mice compared to the vehicle control mice, as illustrated in Supplemental Figure S1A,B.

#### 2.1.2. Systemic Challenge with *T. monococcum* Alcohol-Soluble Gluten Extract Elicits Hypothermic Shock Responses in Skin-Sensitized Mice

Parallel groups of skin-sensitized mice were employed to induce anaphylaxis through systemic challenge with *T. monococcum* alcohol-soluble gluten extract (0.5 mg/mouse) or vehicle. The quantification of anaphylactic reactions was performed by assessing hypothermic shock reactions (HSRs) using rectal thermometry, as detailed in the Methods. No HSR was observed upon vehicle (i.e., zero allergen) or alcohol-soluble gluten extract challenge in control mice (Supplemental Figure S2A,B). In contrast, acute HSRs were evident upon systemic allergen challenge in sensitized mice (Supplemental Figure S2C,D). Significant HSRs were noted from 5 to 30 min post-systemic allergen challenge, as indicated by ANOVA analysis (*p* < 0.05).

#### 2.1.3. Balb/c Mice Exhibit Strong Mucosal Mast Cell Response (MMCR) in Response to Alcohol-Soluble *T. monococcum*-Induced Systemic Anaphylaxis

Supplemental Figure S3A,B depict the MMCR as measured by mucosal mast cell protease (MMCP)-1 response in both control mice and allergic mice. The systemic challenge with *T. monococcum* alcohol-soluble gluten extract, as opposed to the vehicle, leads to a significant elevation in MMCP-1 levels.

### 2.2. Validation of the Transdermal Sensitization and Systemic Elicitation of Disease Mouse Model for Triticum aestivum Using Alcohol-Soluble Gluten Extract

#### 2.2.1. Transdermal Exposure to Alcohol-Soluble Gluten Extract from *T. aestivum* Elicits Robust Specific IgE Antibody Responses in Balb/c Mice

Balb/c female mice were grouped and subjected to transdermal exposure to alcohol-soluble gluten extract from *T. aestivum* (genome AABBDD) or vehicle following a repeated weekly exposure regime outlined in Section 4. Blood samples obtained before the initial exposure (pre) and after the sixth skin exposure (6R) were analyzed for sIgE levels. Remarkably, a significant elevation in sIgE antibody levels was noted after transdermal exposure to alcohol-soluble gluten extract from *T. aestivum*, indicating an increase in allergic mice compared to the vehicle control mice, as depicted in Supplemental Figure S4A,B.

#### 2.2.2. Systemic Challenge with *T. aestivum* Alcohol-Soluble Gluten Extract Elicits Hypothermic Shock Responses in Skin-Sensitized Mice

Groups of skin-sensitized mice were utilized to induce anaphylaxis through systemic challenge with *T. aestivum* alcohol-soluble gluten extract (0.5 mg/mouse) or vehicle. The quantification of anaphylactic reactions was conducted by assessing HSR using rectal thermometry, as outlined in the Methods. No HSR was observed in control mice upon vehicle (i.e., zero allergen) or alcohol-soluble gluten extract challenge (Supplemental Figure S5A,B). Conversely, acute HSRs were evident upon systemic allergen challenge in sensitized mice (Supplemental Figure S5C,D). Significant HSRs were noted from 5 to 30 min post-systemic allergen challenge, as indicated by ANOVA analysis (*p* < 0.05).

#### 2.2.3. Balb/c Mice Exhibit Strong Mucosal Mast Cell Response (MMCR) in Response to Alcohol-Soluble Gluten Extract from *T. aestivum*-Induced Systemic Anaphylaxis

Supplemental Figure S6A,B illustrate the MMCP-1 response in both control mice and allergic mice. It is evident that systemic challenge with *T. aestivum* alcohol-soluble gluten extract, in contrast to vehicle, results in a significant increase in MMCR in the blood.

### 2.3. Mapping the Inherent Allergenicity and Sensitization Potential of Alcohol-Soluble Gluten Extracts Across Diploid, Tetraploid, and Hexaploid Wheat Species

**Figure 1:**
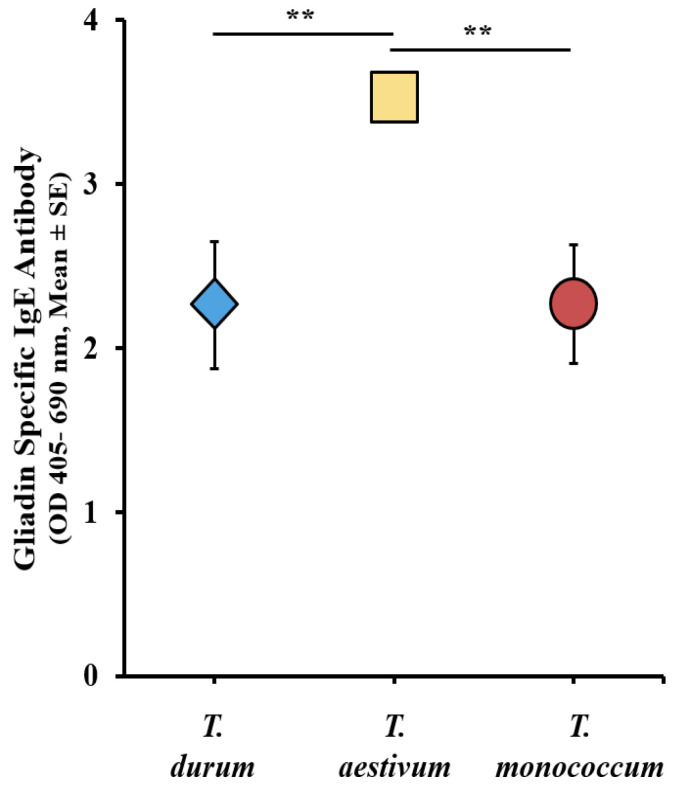
Comparative map of the intrinsic sensitization potentials of alcohol-soluble gluten extract (gliadins) from diploid (*T. monococcum*), tetraploid (*T. durum*), and hexaploid wheats (*T. aestivum*). The change in gliadin-specific IgE antibody levels after the 6th transdermal exposure to alcohol-soluble gluten extracts from the respective depicted wheat species. ** *p* < 0.01, one-way ANOVA and Tukey’s post hoc test. *n* = 10/group. *n*: number of mice. Note: In this figure, the error bar is too small to be visible in one case. For details on the data variance spread, see the Supplemental Figures.

Utilizing the sIgE data from the validation studies mentioned earlier, and our previously reported durum wheat study [33], we constructed a comparative sensitization map. The levels of sIgE antibodies induced by each wheat species were calculated by subtracting the baseline (pre) sIgE levels from the sIgE levels observed after the sixth response (6R). The resulting comparative map illustrates the intrinsic allergenicity and sensitization potential of the three genetically distinct wheat, as depicted in Figure 1. Notably, *T. durum* and *T. monococcum* alcohol-soluble gluten extracts yielded similar sIgE levels, while *T. aestivum* elicited significantly higher sIgE levels.

### 2.4. Comparative Map of the Intrinsic Allergenicity Disease Elicitation Potential of Alcohol-Soluble Protein Extracts from the Diploid, Tetraploid, and Hexaploid Wheats

**Figure 2:**
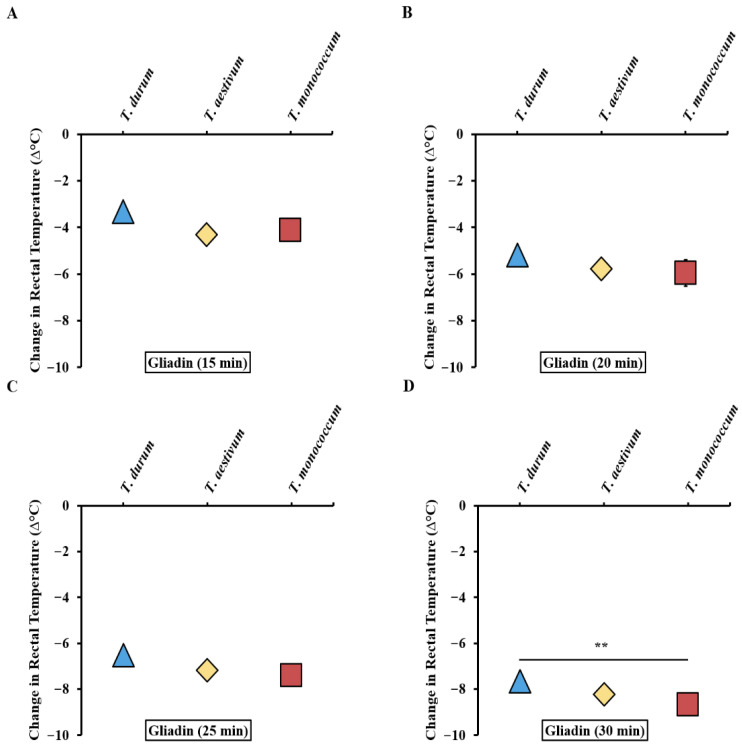
Comparative map of the intrinsic allergenicity disease elicitation potential of alcohol-soluble gluten extracts (gliadins) from tetraploid, hexaploid, and diploid wheat species. (**A**,**B**) HSRs at 15 and 20 min after systemic challenge with 0.5 mg of *T. durum*, *T. aestivum*, and *T. monococcum* respectively. (**C**,**D**) HSRs at 25 and 30 min after systemic challenge with 0.5 mg of *T. durum*, *T. aestivum*, and *T. monococcum* respectively. ** *p* < 0.05, one-way ANOVA and Tukey’s post hoc test. *n* = 10/group. *n*: number of mice. Note: Standard error bars are not visible in many cases. In this figure, error bars are too small to be visible in many cases. For details on the data variance spread, see the Supplemental Figures.

We utilized the absolute changes in rectal temperature data resulting from systemic challenges in the validation studies of *T. durum*, *T. aestivum*, and *T. monococcum*, alongside our previously reported durum wheat study [33] to create a comparative disease elicitation map. Figure 2A–D display the disease elicitation potential map of alcohol-soluble glutens from the three species after 15, 20, 25, and 30 min respectively. As evident, the gliadins from the three wheat species have largely similar reaction kinetic profiles until the 30 min mark (Figure 2D).

### 2.5. Comparative Map of the Mucosal Mast Cell Response (MMCR) Elicitation Potential of Alcohol-Soluble Gluten Extracts from the Diploid, Tetraploid, and Hexaploid Wheats

**Figure 3:**
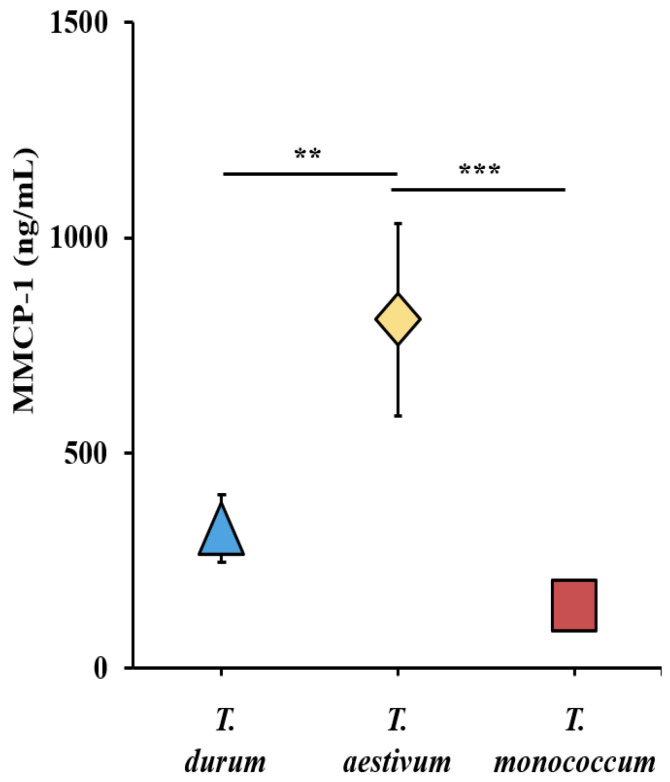
Comparative map of the mucosal mast cell response (MMCR) elicitation potential of alcohol-soluble gluten extracts from diploid, tetraploid, and hexaploid wheats as measured by acute elevation of mucosal mast cell protease (MMCP)-1 levels in the blood. Average MMCP-1 blood level after 0.5 mg systemic allergen challenge dose. ** *p* < 0.05, *** *p* < 0.005, one-way ANOVA and Tukey’s post hoc tests. *n* = 10/group. MMCP-1: mucosal mast cell protease-1. Note: In this figure, error bars are too small to be visible in one case. For details on the data variance spread, see the Supplemental Figures.

We utilized the MMCP-1 data obtained from systemic allergen challenges in the validation studies of *T. durum*, *T. monococcum*, and *T. aestivum* wheats, along with previously reported durum wheat studies [33] to construct a comparative MMCR elicitation potential map. Figure 3 presents the MMCR elicitation potential map at 0.5 mg systemic allergen challenge. It is apparent that alcohol-soluble gluten (gliadin) from *T. aestivum* induced the highest MMCR followed by *T. durum* and *T. monococcum*.

### 2.6. Validation of the Transdermal Sensitization and Systemic Elicitation of Disease Mouse Model for Triticum durum Using Acid-Soluble Gluten Extract

#### 2.6.1. Transdermal Exposure to Acid-Soluble Gluten Extract from *T. durum* Elicits Robust Specific IgE Antibody Responses in Balb/c Mice

Balb/c female mice were grouped and exposed to transdermal application of acid-soluble gluten extract (glutenins) from *T. durum* (genome AABB) or vehicle, following a repeated weekly exposure regimen as outlined in the specified Methods. Blood samples collected before the initial exposure (pre) and after the sixth skin exposure (6R) were analyzed for sIgE levels. Significantly, there was a substantial elevation in sIgE antibody levels after transdermal exposure to acid-soluble gluten extract from *T. durum*, indicating an increase in allergic mice compared to the vehicle control mice, as depicted in Supplemental Figure S7A,B.

#### 2.6.2. Systemic Challenge with *T. durum* Acid-Soluble Gluten Extract Elicits Hypothermic Shock Response (HSR) in Skin-Sensitized Mice

Groups of skin-sensitized mice were employed to induce anaphylaxis through systemic challenge with *T. durum* acid-soluble gluten extract (0.5 mg/mouse) or vehicle. Anaphylactic reactions were quantified using HSR and monitored with rectal thermometry, as detailed in the Methods. No HSR was observed in control mice upon vehicle (i.e., zero allergen), or acid-soluble gluten extract challenge (Supplemental Figure S8A,B). In contrast, acute HSRs were evident upon systemic allergen challenge in sensitized mice (Supplemental Figure S8C,D). Significant HSRs were noted from 5 to 30 min post-systemic allergen challenge, as indicated by ANOVA analysis (*p* < 0.05).

#### 2.6.3. Balb/c Mice Exhibit Strong Mucosal Mast Cell Response (MMCR) in Response to Acid-Soluble *T. durum*-Induced Systemic Anaphylaxis

Supplemental Figure S9A,B illustrate the MMCP-1 responses in both control mice and allergic mice. It is evident that the systemic challenge with *T. durum* acid-soluble gluten extract, as opposed to vehicle, results in a significant increase in MMCP-1 levels in the blood, as depicted.

### 2.7. Validation of the Transdermal Sensitization and Systemic Elicitation of Disease Mouse Model for Triticum monococcum Using Acid-Soluble Gluten Extract

#### 2.7.1. Transdermal Exposure to Acid-Soluble Gluten Extract from *T. monococcum* Elicits Robust Specific IgE Antibody Responses in Balb/c Mice

Balb/c female mice were assigned to groups and subjected to transdermal exposure to acid-soluble gluten extract from *T. monococcum* (genome AA) or vehicle, following a repeated weekly exposure regimen as outlined in Section 4. Blood samples obtained before the initial exposure (pre) and after the sixth exposure (6R) were analyzed for sIgE levels. A significant elevation in sIgE antibody levels was observed following transdermal exposure to acid-soluble gluten extract from *T. monococcum*, indicating an increase in allergic mice compared to the vehicle control mice, as illustrated in Supplemental Figure S10A,B.

#### 2.7.2. Systemic Challenge with *T. monococcum* Acid-Soluble Gluten Extract Elicits Hypothermic Shock Response (HSR) in Skin-Sensitized Mice

Parallel groups of skin-sensitized mice were utilized to induce anaphylaxis through systemic challenge with *T. monococcum* acid-soluble gluten extract (0.5 mg/mouse) or vehicle. The quantification of anaphylactic reactions was conducted by assessing HSR using rectal thermometry, as detailed in the Methods. No HSR was observed upon vehicle (i.e., zero allergen) or acid-soluble gluten extract challenge in control mice (Supplemental Figure S11A,B). In contrast, acute HSRs were evident upon systemic allergen challenge in sensitized mice (Supplemental Figure S11C,D). Significant HSRs were noted from 10 to 30 min post-systemic allergen challenge, as indicated by ANOVA analysis (*p* < 0.05).

#### 2.7.3. Balb/c Mice Exhibit Strong Mucosal Mast Cell Response (MMCR) in Response to Acid-Soluble *T. monococcum*-Induced Systemic Anaphylaxis

Supplemental Figure S12A,B illustrate the MMCR as measured by MMCP-1 responses in both control mice and allergic mice. Upon systemic challenge with *T. monococcum* acid-soluble gluten extract, there is a notable increase in MMCP-1 levels compared to the vehicle, indicating a significant immune response.

### 2.8. Mapping the Inherent Allergenicity and Sensitization Potential of Acid-Soluble Gluten Extracts Across Diploid, Tetraploid, and Hexaploid Wheat Species

**Figure 4:**
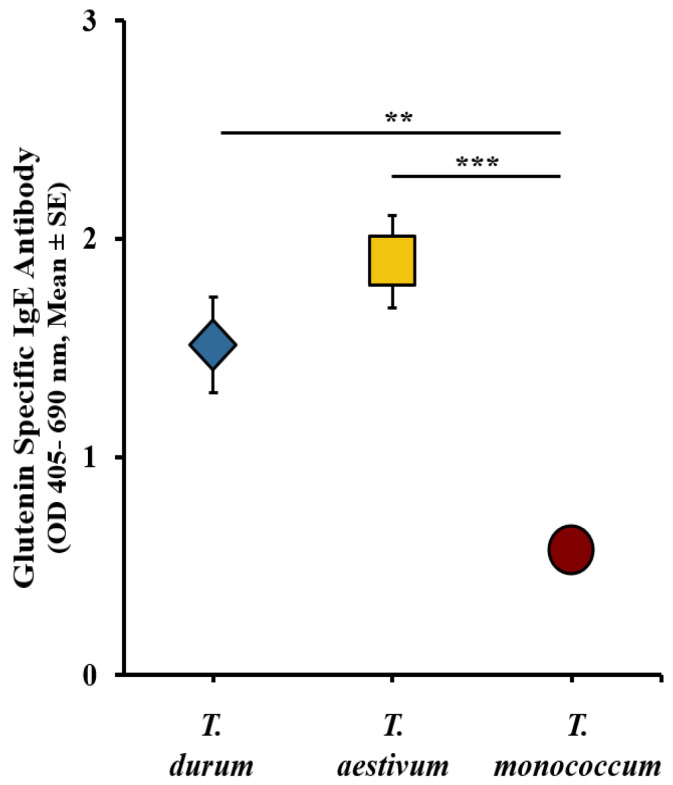
A comparative map of the intrinsic allergenicity sensitization potential of acid-soluble gluten extracts from *T. durum*, *T. aestivum*, and *T. monococcum.* The changes in acid-soluble gluten extract-specific IgE antibody (Ab) levels after the 6th skin exposure to acid-soluble gluten extract from *T. durum*, *T. monococcum*, and *T. aestivum*. ** *p* < 0.05, *** *p* < 0.005 one-way ANOVA and Tukey’s post hoc tests. *n =* 10/group. *n*: number of mice. Note: In this figure, error bars are too small to be visible in one case. For details on the data variance spread, see the Supplemental Figures.

Utilizing the sIgE data from the validation studies mentioned earlier, and our previously reported Ambassador wheat study [34], we constructed a comparative sensitization map. The levels of sIgE antibodies induced by each wheat species were calculated by subtracting the baseline (pre) sIgE levels from the sIgE levels observed after the sixth response (6R). The resulting comparative map illustrates the intrinsic allergenicity and sensitization potential of the three genetically distinct wheat, as depicted in Figure 4. Notably, *T. aestivum* and *T. durum* were significantly elevated when compared to *T. monococcum* acid-soluble gluten extract.

### 2.9. Comparative Map of the Intrinsic Allergenicity Disease Elicitation Potential of Acid-Soluble Gluten Extracts from the Diploid, Tetraploid, and Hexaploid Wheats

**Figure 5:**
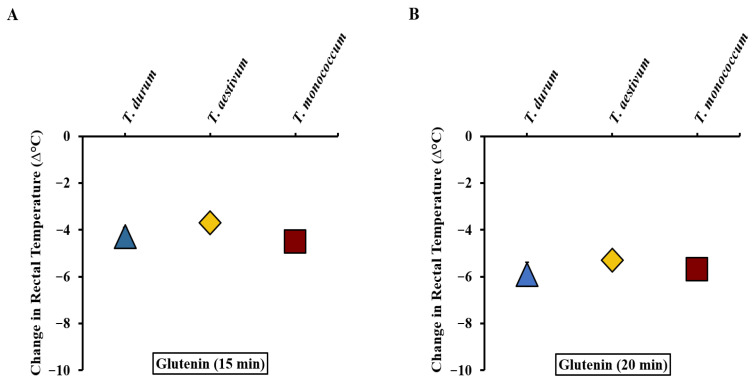

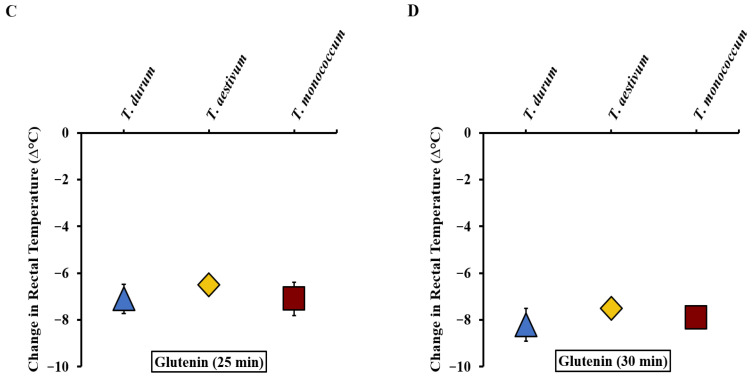
Comparative map of the intrinsic allergenicity disease elicitation potential of acid-soluble gluten extracts from diploid, tetraploid, and hexaploid wheat. (**A**,**B**) HSRs at 15 and 20 min after systemic challenge with 0.5 mg of *T. durum*, *T. aestivum*, and *T. monococcum* respectively. (**C**,**D**) HSRs at 25 and 30 min after systemic challenge with 0.5 mg of *T. durum*, *T. aestivum*, and *T. monococcum* respectively. *n* = 10/group. *n*: number of mice. Note: In this figure, error bars are too small to be visible in some cases. For details on the data variance spread, see the Supplemental Figures.

We utilized the absolute changes in rectal temperature data resulting from systemic challenges in the validation studies of *T. durum*, *T. aestivum*, and *T. monococcum*, alongside our previously reported hexaploid wheat study [34] to create a comparative disease elicitation map. Figure 5A–D display the disease elicitation potential map of the three species after 15, 20, 25, and 30 min respectively. As evident, the three wheat species have similar reaction kinetics through the 30 min period (Figure 5A–D).

### 2.10. Comparative Map of the Mucosal Mast Cell Response (MMCR) Elicitation Potential of Acid-Soluble Gluten Extracts from the Diploid, Tetraploid, and Hexaploid Wheats

**Figure 6:**
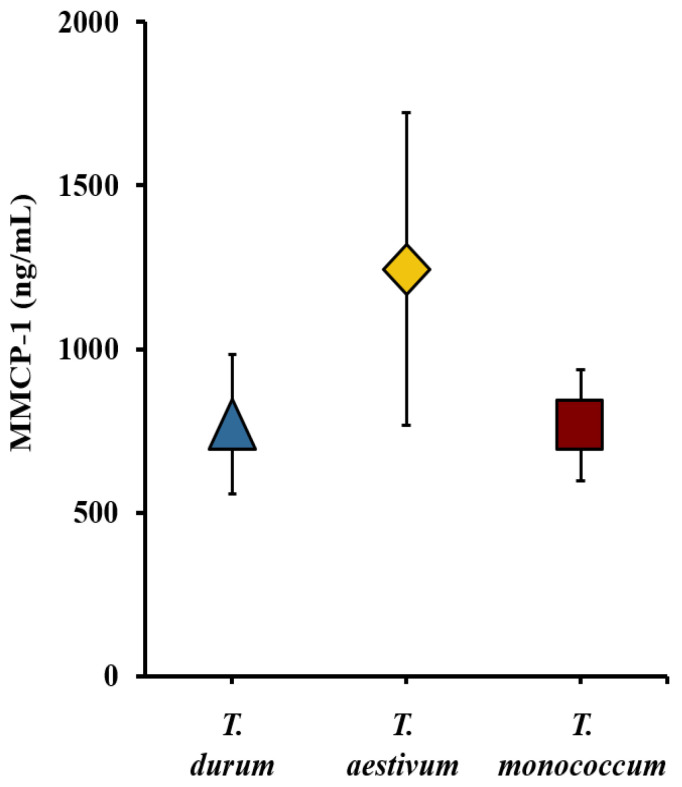
Comparative map of the mucosal mast cell response (MMCR) elicitation potential of acid-soluble gluten extracts from diploid, tetraploid, and hexaploid wheats as measured by acute elevation of mucosal mast cell protease (MMCP)-1 levels in the blood. Average MMCP-1 blood level after 0.5 mg systemic allergen challenge dose. *n* = 10/group. MMCP-1: mucosal mast cell protease-1. *n*: number of mice.

We utilized the MMCP-1 data obtained from systemic allergen challenges in the validation studies of *T. durum*, *T. monococcum*, and *T. aestivum* wheats, along with previously reported *T. aestivum* wheat studies to construct a comparative MMCR elicitation potential map [34]. Figure 6 presents the comparative MMCR elicitation map at 0.5 mg systemic allergen challenge. It is apparent that *T. aestivum* induced the highest MMCR. *T. durum* and *T. monococcum* elicited similar levels of MMCR.

## 3. Discussion

We have organized the discussion as follows: research goals, findings and discussion of the previous relevant published studies, future research directions, and limitations.

*Research goals:* In this study, we sought to develop a comparative map of the intrinsic allergenicity of native glutens from the three commonly cultivated and consumed wheat species. We hypothesized that these three different wheat species will exhibit natural variations in their sensitization capacity as well as their ability to elicit systemic anaphylactic disease. Our results in general support this hypothesis.

In this study, we defined ‘inherent allergenicity’ as the built-in potential of a wheat species to exhibit allergenicity properties (i.e., ability to elicit sensitization via eliciting a specific IgE antibody response and ability to elicit clinical anaphylaxis reactions as measured by hypothermic shock responses) in our mouse model that does not use external adjuvants such as alum or detergents. We defined ‘sensitization potential’ as the built-in potential of a wheat species to elicit a specific IgE antibody response upon skin exposure in this mouse model.

*Findings and discussion of the previous relevant published studies:* This study yielded eight novel findings, encompassing: (i) the first-time validation of the model for intrinsic allergenicity of alcohol-soluble gluten (gliadin) extracts from two commercially cultivated wheat species namely *T. aestivum* (common bread wheat hexaploid of AABBDD genome) and *T. monococcum* (ancient diploid einkorn wheat of AA genome); (ii) creation of the first comparative map of intrinsic sensitization potential of alcohol-soluble gluten extracts from *T. aestivum*, *T. monococcum*, and *T. durum*; (iii) establishment of a comparative map showcasing the intrinsic hypothermic shock response (HSR) elicitation potential of alcohol-soluble gluten extracts from the three commonly cultivated wheat species; (iv) development of a comparative map highlighting the mucosal mast cell response elicitation potential of alcohol-soluble gluten extracts from the three commonly cultivated wheat species; (v) the first-time validation of the model for intrinsic allergenicity of acid-soluble gluten (glutenin) extracts from two commercially cultivated wheat species namely *T. durum* (tetraploid of AABB genome) and *T. monococcum* (ancient diploid einkorn wheat of AA genome); (vi) creation of the first comparative map of intrinsic sensitization potential of acid-soluble gluten extracts from *T. aestivum*, *T. monococcum*, and *T. durum*; (vii) establishment of a comparative map showcasing the intrinsic hypothermic shock response (HSR) elicitation potential of acid-soluble gluten extracts from the three commonly cultivated wheat species; and (viii) development of a comparative map highlighting the mucosal mast cell response (MMCR) elicitation potential of acid-soluble gluten extracts from the three commonly cultivated wheat species.

Previously, we had developed and characterized the transdermal sensitization and systemic disease elicitation mouse model using the alcohol-soluble gluten extract (gliadin) from *T. durum*, and the acid-soluble gluten extract (glutenin) from *T. aestivum* [33,34]. In this study, we further validated this model for alcohol-soluble gluten extracts from *T. aestivum* and *T. monococcum* and acid-soluble gluten extracts from *T. durum* and *T. monococcum*. We chose these three wheat species because of the following reasons: (i) *T. aestivum* wheat (variety Ambassador, a hexaploid AABBDD genome) is the common wheat primarily employed in cracker and cookie production [35]; (ii) *T. durum* wheat is the tetraploid genome (AABB genome) is the common wheat primarily used for making pastas such as spaghetti or penne [36]; and (iii) *T. monococcum* (known as einkorn, AA genome) is a commercially available ancient wheat species with considerable research interest in its potential health-promoting properties [37].

In this study, the order of sensitization capacity for three wheat species were as follows: (i) for gliadins, *T. aestivum* > *T. durum* = *T. monococcum*; (ii) for glutenin, *T. aestivum* > *T. durum* > *T. monococcum*. There are no previous studies comparing the allergenic sensitization capacity of gluten proteins in mice. There is one previous study comparing the sensitization capacity of non-gluten proteins in mice [38]. They found the order of sensitization capacity for the three wheat species as follows: *T. aestivum* ≥ *T. durum* > *T. monococcum*. Thus, these studies together suggest that *T. monococcum* has the least sensitization capacity independent of the type of allergen. In contrast, *T. aestivum* has the highest sensitization capacity.

There are two studies conducted using IgE from wheat-allergic human subjects to investigate the relative allergenicity of different wheat species/varieties. Nakamura et al. 2005 used urea-soluble wheat allergens that included both gluten (gliadin and glutenin) and non-gluten proteins extracted from 321 wheat varieties belonging to various species as follows: (*T. monococcum*), tetraploid (*T. durum*, *T. dicoccum*, *T. polonicum*, *T. turgidum*), and hexaploid (*T. aestivum*, *T. compactum*, *T. spelta*) [20]. They reported that einkorn was among the least allergenic wheats and *T. aestivum* was the highest. Larre et al. 2011 compared non-gluten proteins’ allergenicity from two wheat species: *T. monococcum* (Engrain) and *T. aestivum* (cv Recital) [39]. They used IgE ELISA for testing using serum from 20 wheat-allergic subjects. They reported that the majority of patient’s IgE reacted most with *T. aestivum* and least with *T. monococcum*. Thus, in humans, einkorn appears to be the least potent sensitizer and *T. aestivum* appears to be the most prolific sensitizer for allergic reactions. These results in principle are consistent with mouse model studies on gluten as well as non-gluten allergenicity with *T. monococcum* being the lowest sensitizer and *T. aestivum* being the greatest sensitizer. Therefore, the adjuvant-free mouse model used in our studies appears to mimic human sensitization capacities of different wheat species. These findings further support the utility of this novel mouse model to predict the human allergenicity of novel wheat proteins in the future.

Previous studies reviewed as above used polysensitized plasma samples obtained from allergic subjects who were expected to be naturally sensitized to multiple wheats (i.e., polysensitization exposure). Therefore, they could test various wheat varieties and conclude their results as relative allergenicity variations based on specific IgE binding to coated proteins from various wheats. In contrast, our mouse model is a mono-sensitization model, wherein different groups of mice are sensitized to different single wheat species. Therefore, in our model, only mono-sensitized plasma obtained from mice sensitized to a particular wheat species was tested for specific IgE antibody levels against that wheat species. Therefore, our constants are the mouse model parameters used for skin exposure with identical exposure doses (1 mg per skin application in 100 μL) across all wheat species. Furthermore, we also used identical doses of protein (1 mg/mL, 50 μL per well) to coat in ELISA as detailed in Section 4. Thus, the insight we obtained in this study was as follows: the glutens obtained from different wheat species have different intrinsic capacities to elicit specific IgE antibody responses upon skin exposure at identical doses, the likely reason underling the observed differences being that they contain different types/amounts of gluten allergenic components that are capable of eliciting specific IgE responses when exposed to the skin at identical doses; detailed studies characterizing such differences among wheat species are clearly extremely important but are beyond the scope of this current study—a topic for future research.

The gluten allergy mechanism occurs in two distinct phases. In the initial stage, genetically predisposed individuals encounter gluten through various routes, such as the skin, gut, eyes and airways. This exposure triggers the production of gluten-specific IgE antibodies [40,41,42]. These antibodies subsequently bind to mast cells and basophils through the high-affinity IgE receptor (FcϵRI), thus establishing a sensitized immune state. The second phase unfolds upon re-exposure to gluten in sensitized individuals. Gluten allergens bind to IgE-loaded mast cells and basophils, initiating the release of chemical mediators that culminate in clinical manifestations, including systemic anaphylaxis [5]. To assess the sensitization potential of glutens from the three wheat species used in this study, we employed a highly sensitive ELISA-based method, as previously described [33,34,38]. Our findings reveal that both types of gluten extracts from all three species inherently possess the capability to induce sensitization through skin exposure.

The HSR serves as an indicator of the impact of anaphylaxis on the neurological and cardiovascular functions associated with thermoregulation. Employing rectal thermometry, HSR is a widely utilized method to assess the severity of anaphylaxis in mouse models of food allergy, as it is considered a surrogate marker for cardiac output, which is adversely affected during food-induced systemic anaphylactic reactions [33,34,38,43,44]. Notably, in mouse models of anaphylaxis, HSR can arise from both IgE and IgG-mediated reactions following intraperitoneal challenge [45,46]. Our findings demonstrate that the HSR can effectively quantify systemic anaphylaxis induced by both gluten extracts from all three wheat species in this mouse model.

Previously identified as a specific biomarker for IgE-antibody-mediated systemic anaphylaxis in mouse models of food allergy, MMCP-1 has been established as a reliable indicator [47]. Given that HSR reflects both IgE and IgG1-mediated systemic anaphylaxis, we opted to incorporate MMCP-1 as an additional biomarker. This allowed us to specifically assess whether glutens from the three wheat species can instigate life-threatening anaphylaxis through the IgE pathway, and whether there are distinctions in the capacity of the two gluten types to initiate the IgE-mediated pathway of disease elicitation. It is crucial to note that MMCP-1 is exclusively sourced from mucosal mast cells present in the gut. These gastrointestinal mucosal mast cells contain MMCP-1 within granules, a unique protein absent in connective tissue mast cells [48]. These mast cells in the gut possess receptors for IgE antibodies. Following sensitization to gluten, IgE antibodies attach to mucosal mast cells and basophils through the FcϵRI. In pre-challenge samples, blood exhibits minimal MMCP-1 levels, as the mucosal mast cells remain inactive. However, upon gluten challenge, the gluten binds to IgE antibodies on the gut mast cells, triggering mast cell activation and degranulation. This process releases MMCP-1 into the systemic circulation. Therefore, MMCP-1 levels one hour after challenge serve as an indicator of IgE-antibody-mediated anaphylaxis in the gut, simulating the natural IgE-mediated reactions observed in gluten allergy upon ingestion of gluten-containing products. Our data reveals that both gluten extracts can activate this pathway, with the acid-soluble gluten extract showing a slightly superior capacity to elicit MMCP-1 release.

A goal of this study was to evaluate the anaphylaxis potential of glutens from common wheat species upon chronic skin exposure. Therefore, we studied anaphylaxis after nine exposures which we consider as the chronic exposure protocol. The responses we noted are indeed too severe reflecting the inherent capacity of glutens to elicit life-threatening anaphylaxis in this model. We were required to have at least 2 weeks’ time interval between the last skin exposure and systemic challenge to elicit anaphylaxes per our approved animal protocol because of a blood collection time interval requirement in this animal species.

In this study, we used a highly sensitive ELISA method to measure gluten-specific IgE antibody levels that we have reported before [33,34]. This ELISA method was a modified method of a previously reported method by us that was comparable to the rat-based passive cutaneous anaphylaxis in vivo assay to measure food-specific IgE antibody; in this ELISA method, food-specific IgE antibodies can be measured even when IgG1 is present [49]. Furthermore, we also found that total IgE measurements that do not require wheat protein coating and that are not affected by IgG correlate positively with the specific IgE measurements as reported [33,34]. This is further illustrated by the similar pattern of specific IgE comparative sensitization map as well as total IgE comparative sensitization map shown for glutenin (Supplemental Figure S13).

*Future research directions:* Numerous studies indicate that the allergenicity of food proteins, including those found in wheat, can be influenced by food processing [6]. For example, employing novel processing methods on wheat allergens may lead to the generation of new epitopes or the revelation of previously concealed epitopes, potentially heightening their allergenicity. The proposed methodology facilitates the development of intrinsic allergenicity maps using processed wheat. By comparing the allergenicity potential maps of native and processed wheat proteins, it becomes feasible to quantify the effects of processing on intrinsic allergenicity within each wheat genotype. In essence, creation of the first gluten allergenicity will assist the development of novel potentially hypoallergenic glutens. It also helps to prevent inadvertent introduction of hyperallergenic processed glutens into food and cosmetic products.

**Figure 7:**
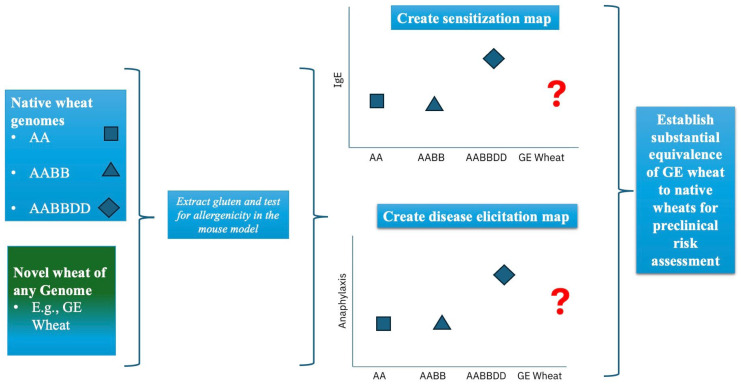
Proposed conceptual framework to use the comparative gluten allergenicity map to establish substantial equivalence of novel wheat glutens. A novel gluten obtained from, for example, a genetically engineered (GE) wheat line can be tested for intrinsic allergenicity using the mouse model. Following quantification of sensitization capacity and disease elicitation capacity, its potency can be mapped relative to the native wheat line of a particular species as well as other commonly used wheat species of different genomes as shown. Using the quantitative data and statistical methods, substantial equivalence of the GE gluten to the native gluten can be established. A similar approach may also be used to establish substantial equivalence of physically or chemically altered novel glutens to native glutens. Conducting dose–response studies for both sensitization and disease elicitation, as well as determining the exposure time-course impact on such dose–response curves, will be critical to make meaningful interpretations of the data. Thus, this comparative tool will assist in determining the hypo-/hyper-/iso-allergenicity phenotype status of novel wheats.

Currently, GE wheat is not available for consumption in the United States. However, the US FDA recently approved a GE wheat produced by Argentina and Brazil [21,50]. GE wheat has recently been approved for eating and growing in Argentina and Brazil, and it has been approved for consumption in Australia, New Zealand, and Nigeria [50]. However, the allergenic potential of this GE wheat or other such novel wheats must be carefully evaluated to establish whether they may be hyper-/hypo-/non-allergenic compared to their common non-GE counterparts. The methodology described here can be used for this purpose. The comparative map will serve as a tool to objectively quantify the relative allergenicity potential of GE wheat versus non-GE native counterparts and establish substantial equivalence. A conceptual framework of how the gluten allergenicity map created here may be used to establish substantial equivalence of novel glutens is shown in Figure 7. By comparing the allergenicity potential maps of the novel wheat lines versus the traditional non-GE wheat, it becomes feasible to quantify the impact of genetic modification/engineering on intrinsic allergenicity of wheat—a major long-standing concern for GE foods.

It is important to note that we selected one representative variety from each of the three wheat species for this study, because of the limited resources available. Further research is necessary to validate whether the chosen variety adequately represents the intrinsic allergenicity potential of all/most other varieties within the species [51]. This same methodology could be employed to investigate the differences in intrinsic allergenicity potential between various wheat lines/varieties. This approach has the potential to identify hypoallergenic and non-allergenic wheat lines by utilizing comparative mapping. In the same vein, by utilizing comparative potential maps of allergenicity, it is also possible to identify and exclude potentially hyperallergenic wheat lines, thus safeguarding individuals sensitive to wheat allergens.

An additional important issue to consider for future application of the proposed use of the gluten allergenicity map is the assessment of novel GE wheats. The difference in IgE antibody levels might depend largely on the content of known gluten allergen components within each gluten extract under testing; therefore, if a novel allergen were to appear in a GE wheat, it may not be possible to detect variations in IgE antibody levels caused by that specific allergen using only a crude extract of the GE wheat. To address this issue, investigators may consider quantifying the gliadin or glutenin components derived from each wheat variety using Western blotting or ELISA and then confirm their correlation with the variations in IgE antibody levels observed in their studies.

The overall goal of this study was to create a comparative gluten allergenicity map. It is very important to note that the exploration of the molecular mechanisms underlying the differences in allergenicity reported in this study is unclear at present. Molecular information on the differences in the number, structure, and IgE-binding affinity of allergenic epitopes, as well as information on differences in immune signaling pathways (e.g., Th2, Th17, Th1 cytokines, chemokines, etc.) activated by different wheat glutens, is vital to explain the underlying mechanisms. Such detailed mechanisms of study are the suggested future direction of research.

*Limitations of the study:* For these studies, we produced our own gluten-free mice. We used adult mice of 6–8-week age in this study. We had to use mice over this age, because technically it is extremely difficult and too expensive to produce and use an adequate number of mice all belonging to a single week’s age (for example, all belonging to 6 weeks age). Furthermore, our overall goal in this study was to establish a comparative gluten allergenicity map using adult mice; our goal was not to study week-wise effects of age. Therefore, this age variation in the mice used represents a limitation of this study.

It is noteworthy that by using specific IgE antibody absorbance values alone, it will be difficult to interpret the results about the intensity of allergenicity of novel wheats as hyper-, hypo- or non-allergenic compared to the conventional wheats—this is another limitation of this study. However, based on additional testing of the total IgE levels elicited, which measures the absolute quantity of elicited total IgE in the blood as ng/mL that correlates positively with specific IgE antibody absorbance values in the mouse model, we propose the hypothesis that it may be possible to estimate the sensitization potentials of novel wheat extracts comparative to the conventional ones. Furthermore, to evaluate the allergenicity intensity of each wheat extract more accurately, one can also consider making additional comparisons using a standard wheat extract and plasma from mono-sensitized mice.

It is noteworthy that in the present study, a chronic skin exposure protocol was used to develop the life-threatening anaphylaxis as measured by HSR with a dramatic drop in rectal temperature upon intraperitoneal allergen challenge at the optimized single dose of the allergen as presented in this study. However, a single dose challenge testing resulting in such dramatic HSR will not be useful to classify novel wheats as hypo-, hyper- or non-allergenic. To address this issue, it is important to note that the intensity of rectal temperature drop is dependent upon the dose of the allergen used in the systemic challenge. Therefore, for relative comparison of a novel GE wheat with conventional wheats, it is critical to conduct side-by-side dose–response studies (for both sensitization as well as disease elicitation) and develop dose–response curves to make final determination on hypo-, hyper- or non-allergenicity assessments of GE wheats. Furthermore, one can also evaluate the impact of exposure duration (e.g., two skin exposures vs. six skin exposures vs. nine skin exposures) on those dose–response curves for sensitization as well as for disease elicitation. Such comprehensive dose–response studies will be critical to make final determination on hypo-, hyper-, or non-allergenicity interpretation of GE wheats compared to the conventional non-GE wheats (Figure 7). The lack of our ability at present to conduct such dose–response studies is a limitation of this study.

## 4. Materials and Methods

### 4.1. Chemicals and Reagents

Biotin-conjugated antibodies specific to mouse IgE were obtained from BD Bio-Sciences (San Jose, CA, USA). The p-nitrophenyl phosphate compound was sourced from Sigma (St. Louis, MO, USA), and streptavidin alkaline phosphatase was acquired from Jackson ImmunoResearch (West Grove, PA, USA). Folin reagent was obtained from Bio-Rad (Hercules, CA, USA). Specific reagents, including the IgE Mouse Uncoated ELISA Kit with Plates, streptavidin-HRP, TMB substrate, MCPT-1 (MMCP-1) Mouse Uncoated ELISA Kit with Plates, avidin-HRP, and TMB substrate, were procured from Invitrogen/Thermo Fisher Scientific (Waltham, MA, USA).

### 4.2. Mice Breeding and Establishment of a Plant-Protein-Free Mouse Colony

Breeder pairs of adult Balb/c mice were procured from The Jackson Laboratory (Bar Harbor, ME, USA). Upon their arrival, the mice were introduced to a stringent plant-protein-free diet (AIN-93G, Envigo, Madison, WI, USA). After a one-week acclimation period, conventional breeding methods were employed to initiate reproduction. Female mice aged 6–8 weeks from the litters were used in this study. Throughout the entire research period, all mice were consistently maintained on the strict plant-protein-free diet (AIN-93G). All animal procedures strictly adhered to the guidelines established by Michigan State University.

### 4.3. Preparation of Gluten Extract from Wheat Flour

The following wheat species were used to obtain gluten extracts in this study: *T. monococcum* (einkorn, genome AA), *T. durum* (durum wheat, Carpio variety, genomes AABB), *T. aestivum* (common wheat, Ambassador variety, genomes AABBDD) wheats; the *T. aestivum* (Ambassador variety) and *T. durum* (Carpio variety) were obtained from the Michigan State University Wheat Breeding Program and North Dakota State University respectively; and the *T. monococcum* (einkorn wheat) was purchased from a commercial source (URL: www.einkorn.com/ accessed on 1 January 2016).

Protein extraction from wheat flour targeted the isolation of alcohol- and acid-soluble wheat gluten using Osborne’s sequential extraction method [52]. Briefly, a mixture of flour and filter-sterilized 0.5 M NaCl (1:10, *m*/*v*) underwent continuous agitation for 2 h, followed by centrifugation at 20,000× *g* for 30 min. The resulting pellets were preserved for alcohol extraction. The salt-insoluble pellets were then mixed in a 1:10 ratio with 70% ethanol for 2 h, and centrifuged at 20,000× *g* for 15 min, yielding the alcohol-soluble gluten extract supernatant and alcohol-insoluble pellets. The latter was saved for acid extraction. The alcohol-insoluble pellets were combined in a 1:4 ratio with 0.05 M acetic acid for two hours and centrifuged at 20,000× *g* for 15 min. The resulting supernatants from both extractions were frozen at −70 °C overnight and subjected to freeze-drying the next day. The lyophilized alcohol-soluble gluten extract was reconstituted using 70% ethanol and the acid-soluble gluten extract was reconstituted using 0.05 M acetic acid to achieve a concentration of 1 mg protein per 100 µL for topical application. For challenges involving intraperitoneal (IP) injections, the gluten extracts were reconstituted with phosphate-buffered saline (PBS) to attain concentrations of 0.5 mg/mouse. Protein content was quantified using the LECO total combustion method from LECO (St. Joseph, MI, USA). SDS-PAGE testing was conducted to assess protein quality.

### 4.4. Skin Sensitization, Bleeding, and Plasma Sample Preparation

Female adult Balb/c mice were utilized for experimental purposes. To facilitate the procedures, the hair on the mice’s rumps was bilaterally removed using a Philips hair clipper (Amsterdam, The Netherlands). The wheat glutenin was administered onto the rump at a dosage of 1 mg per mouse per application. The transdermal sensitization protocol we have developed involves applying 100 μL of the test material over the rump skin (50 μL per rump) using smooth pipette tips and gently spreading it over the skin area and allowing it dry quickly. There was no anesthesia used in this protocol. In this optimized method that we have used for more than 2 decades, there is no risk of running off material as the volume is very small.

Following application, the treated area was covered with a non-latex bandage sourced from Johnson & Johnson (New Brunswick, NJ, USA) and left in place for one day. This process was repeated on a weekly basis, occurring nine times over a span of nine weeks. Blood samples were collected from the saphenous vein before the initial exposure and after the sixth exposure. The blood was drawn into tubes coated with the anticoagulant lithium heparin (Sarstedt Inc. MicrovetteCB 300 LH, Nümbrecht, Germany). The collected blood was subsequently subjected to centrifugation to isolate plasma, which was then stored individually at −70 °C until required for subsequent testing of sIgE.

### 4.5. Elicitation of Systemic Anaphylaxis

Two weeks after the final cutaneous exposure to alcohol or acid-soluble gluten extract or the vehicle, the mice underwent an IP injection. This injection involved either 0.5 mg of alcohol or acid-soluble gluten extract. Phosphate-buffered saline (PBS) was used as a vehicle for the systemic challenge. Glutenin dissolves completely in PBS; however, gliadin requires slow mixing and typically forms a suspension. We administered 100 μL of the PBS solution containing the respective glutens to elicit anaphylaxis.

### 4.6. Determination of Hypothermic Shock Responses as a Quantifiable Marker of Systemic Anaphylaxis

Rectal temperature (°C) measurements were recorded both before the challenge and at 5 min intervals following the challenge, up to a 30 min duration. A rectal thermometer (DIGI-SENSE, Thermo Fischer Scientific, Waltham, MA, USA) was used for these measurements. The recorded values included the specific temperatures, and the corresponding differences (∆°C) compared to the pre-challenge temperatures for each individual mouse. These recorded data points were subsequently utilized for further analyses.

### 4.7. Measurement of Specific IgE Antibody Levels

Gluten-specific (s) IgE antibody levels were quantified using a highly sensitive ELISA method, as previously detailed with certain modifications [19,33,34,51]. This ELISA method has been optimized such that food-specific IgE can be measured even in the presence of IgG1 antibodies [33,34,51]. For protocol details, readers are to be referred to our previously published method paper [51]. Initially, 96-well Corning 3369 plates were coated with the glutens (1 mg/mL of coating buffer, 50 μL per well) and subsequently blocked using a 5% gelatin solution. After a thorough washing step, plasma samples were introduced onto the plate. Further washing ensued, followed by the addition of a biotin-conjugated anti-mouse IgE antibody. Subsequent washes were performed before introducing streptavidin alkaline phosphatase, and eventually, p-nitrophenyl phosphate was added to enable quantification, following established methodologies [19,33,34,49,51,53,54].

### 4.8. Measurement of Total Plasma IgE Concentration

Plasma levels of total IgE concentrations were determined utilizing an ELISA method based on a commercial test kit as described before [33,34] (Invitrogen ELISA kit, Waltham, MA, USA). In brief, ELISA plates were coated with a capture antibody, followed by plasma samples and then an anti-mouse IgE was used as a secondary antibody followed by a detection system of streptavidin-HRP and TMB substrate as described by previous studies.

### 4.9. Quantification of Mucosal Mast Cell Protease-1 (MMCP-1) Level

One-hour post-challenge, blood samples were obtained and employed to measure the levels of MMCP-1 in the plasma. The quantification was performed using an ELISA-based method developed by Invitrogen, following established procedures [33,34]. In detail, 96-well Corning Costar 9018 plates were initially coated with a capture antibody (anti-mouse MMCP-1). Subsequently, both samples and standards (recombinant mouse MMCP-1) were added to the plate. A biotin-conjugated anti-mouse MMCP-1 antibody was then introduced as the secondary antibody. Detection was achieved using an avidin-HRP/TMB substrate system. It is noteworthy that the assay has a limit of detection set at 120 pg/mL, and the range of standards covered from 120 to 15,000 pg/mL. Testing was conducted in quadruplicate for each individual mouse’s plasma.

## 5. Conclusions

For the first time, we illustrate similarities and distinctions in the inherent allergenic properties among the three chosen genetically distinct wheat species. This preclinical comparative mapping approach could serve in identifying potentially hyperallergenic, hypoallergenic, and non-allergenic wheat varieties created through crossbreeding and genetic engineering. Additionally, it also offers a means to evaluate alterations in wheat protein allergenicity resulting from various physical and chemical processing methods.

## Data Availability

The original contributions presented in this study are included in the article/Supplementary Materials. Further inquiries can be directed to the corresponding author.

## Supplementary Materials

The following supporting information can be downloaded at https://www.mdpi.com/article/10.3390/ijms27093716/s1.

## Abbreviations

The following abbreviations are used in this manuscript:

AA	Genome of Diploid Wheat
AABB	Genome of Tetraploid Wheat
AABBDD	Genome of Hexaploid Wheat
AIN-93G	American Institute of Nutrition in 1993 Growing Rodent diet
ANOVA	Analysis of Variance
CD	Celiac Disease
CGAM	Comparative Gluten Allergenicity Map
ELISA	Enzyme Linked Immuno Sorbent Assay
FAO	Food and Agricultural Organization
GE	Genetically Engineered
HRP	Horse Radish Peroxidase
HSR	Hypothermic Shock Response
IP	Intraperitoneal
MMCR	Mucosal Mast Cell Response
MMCP-1	Murine Mast Cell Protease-1
NCGS	Non-Celiac Gluten Sensitivity
PBS	Phosphate-Buffered Saline
sIgE	Specific IgE antibody
TMB	3,3′, 5,5;-Trimethyl benzidine
US FDA	United States Food and Drug Administration
WHO	World Health Organization
