# Supplementary material for: Creation of the First Comparative Gluten Allergenicity Map Using a Mouse Model: A Preclinical Tool to Establish Substantial Equivalence of Novel Wheat Glutens
Source: Int J Mol Sci. 2026 Apr 22;27(9):3716. doi: 10.3390/ijms27093716 (PMC13163998; doi:10.3390/ijms27093716)
Supplement: Supplementary file 1 [file ijms-27-03716-s001.zip › ijms-4133919-supplementary.pdf]

Supplemental Figures S1-S13 in Jorgensen et al 2026

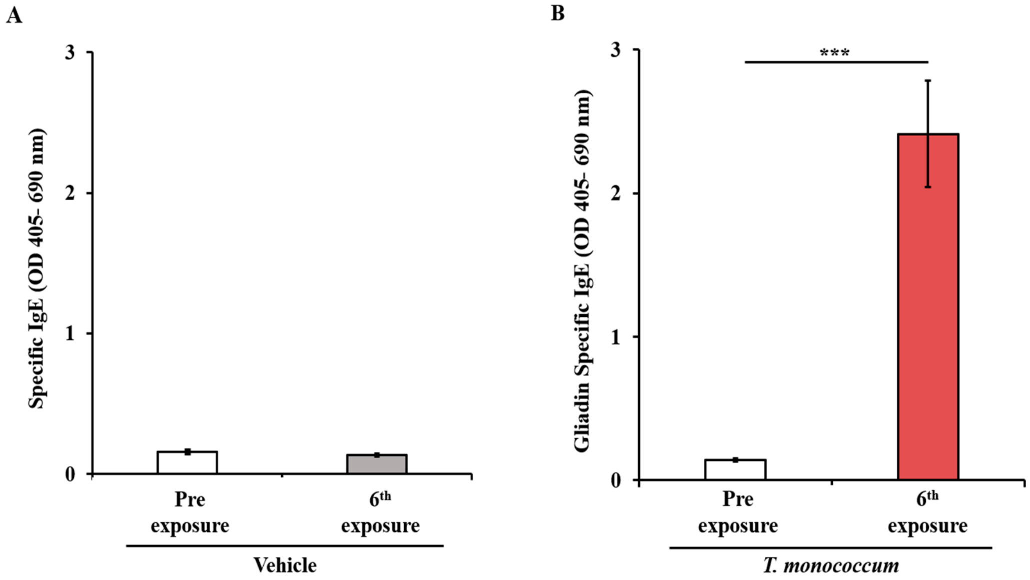

Figure S1. Transdermal exposure of Balb/c mice to alcohol-soluble gluten extract from *T. monococcum* (genome AA) elicited robust specific (s) IgE antibody responses.

Mice were exposed to alcohol-soluble gluten extract from *T. monococcum*, or vehicle as described in Methods. Plasma collected before the 1st exposure (Pre) and after the 6th expo-sure (6R) was used in the measurement of sIgE levels (OD 405–690 nm), with a sample size of n = 10/group. (A) sIgE levels in control mice. (B) sIgE levels in sensitized mice. *** $p < 0.001$, student's t-test. Ab: antibody; *n*: number of mice; OD: optical density.

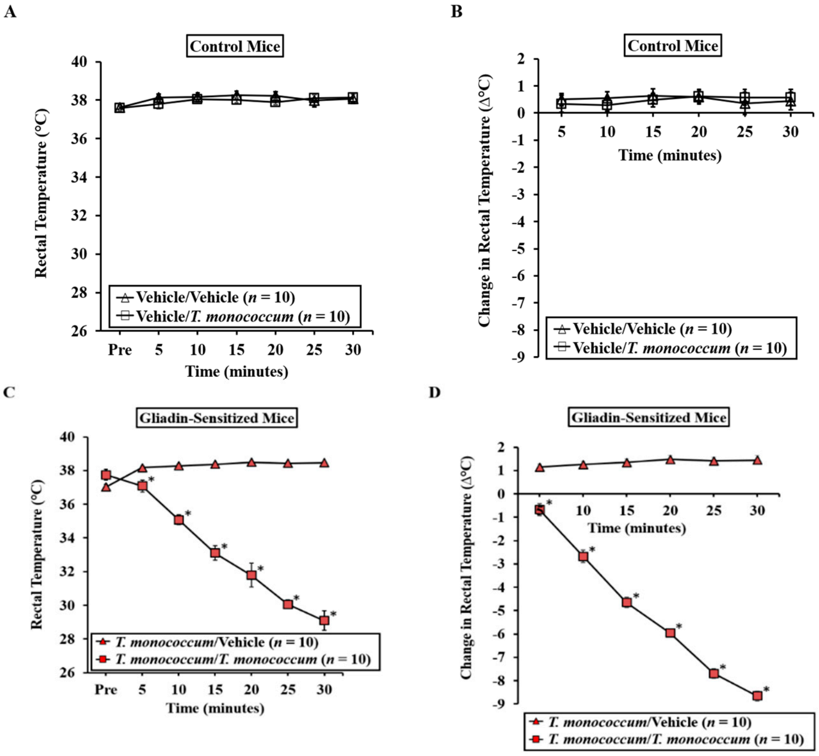

Figure S2. Transdermal sensitization with alcohol-soluble gluten extract from *T. monococcum* is sufficient for eliciting systemic anaphylaxis in Balb/c mice.

Mice were sensitized and systemically challenged with alcohol-soluble gluten extract from *T. monococcum* or vehicle, as described in Materials and Methods. n = 10/group. (A) Actual rectal temperature at indicated time points in control mice challenged with alcohol-soluble gluten extract from *T. monococcum* or vehicle. (B) Change in rectal temperature at indicated time points in control mice challenged with alcohol-soluble gluten extract from *T. monococcum* or vehicle. (C) Actual rectal temperature at indicated time points in alcohol soluble *T. monococcum* protein extract-sensitized mice challenged with alcohol-soluble *T. monococcum* protein extract or vehicle. (D) Change in rectal temperature at indicated time points in alcohol-soluble gluten extract from *T. monococcum*-sensitized mice challenged with alcohol-soluble gluten extract from *T. monococcum* or vehicle. *$p < 0.05$, ANOVA; *n*: number of mice.

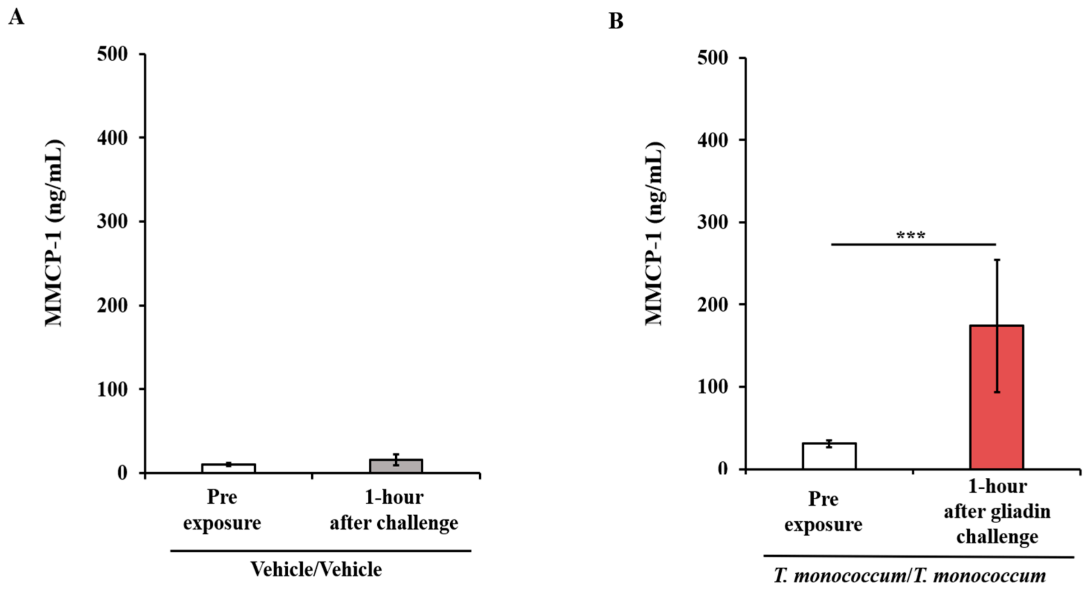

Figure S3. Systemic challenge with alcohol-soluble gluten extract from *T. monococcum* elicits a robust mucosal mast cell response (MMCR) in Balb/c mice.

Mice were sensitized and systemically challenged with alcohol-soluble gluten extract from *T. monococcum* or vehicle, as described in Materials and Methods. Plasma levels of mucosal mast cell protease (MMCP)-1 (ng/mL) in pre and one-hour after challenge were measured via ELISA. n =10/group. (A). MMCP-1 levels in control mice challenged with vehicle. (B) MMCP-1 levels in allergic mice challenged with alcohol-soluble gluten extract from *T. monococcum*. *** $p < 0.001$, student's t-test; *n*: number of mice.

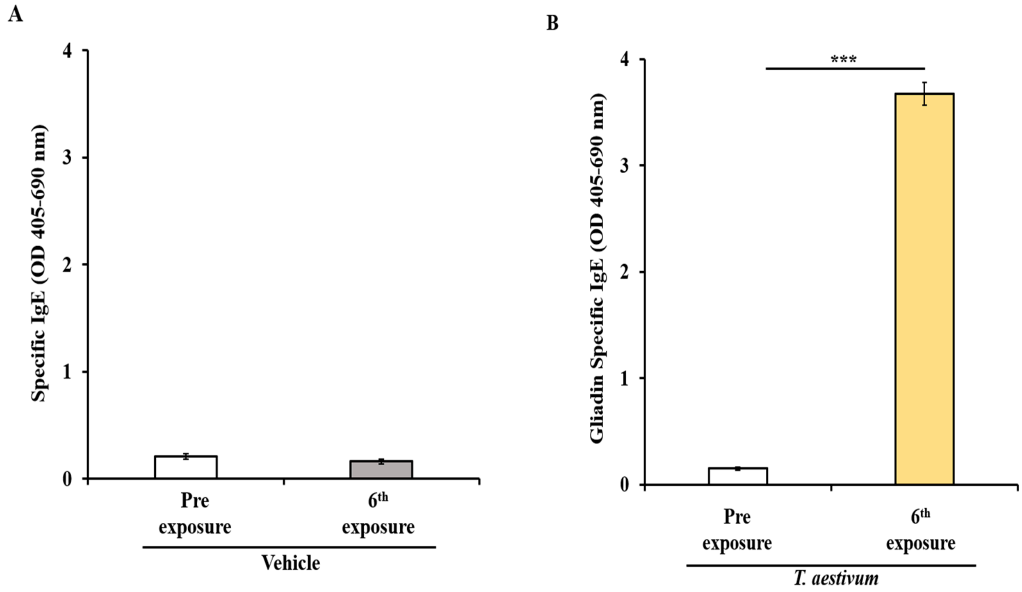

Figure S4. Transdermal exposure of Balb/c mice to alcohol-soluble gluten extract from *T. aestivum* (genome AABBDD) elicited robust specific (s) IgE antibody responses.

Mice were exposed to alcohol-soluble gluten extract from Triticum aestivum or vehicle as described in Methods. Plasma collected before the 1st exposure (Pre) and after the 6th exposure (6R) was used in the measurement of sIgE levels (OD 405–690 nm), with a sample size of n = 10/group. (A) sIgE levels in control mice. (B) sIgE levels in sensitized mice. *** $p < 0.001$, student's t-test. Ab: antibody; *n*: number of mice; OD: optical density.

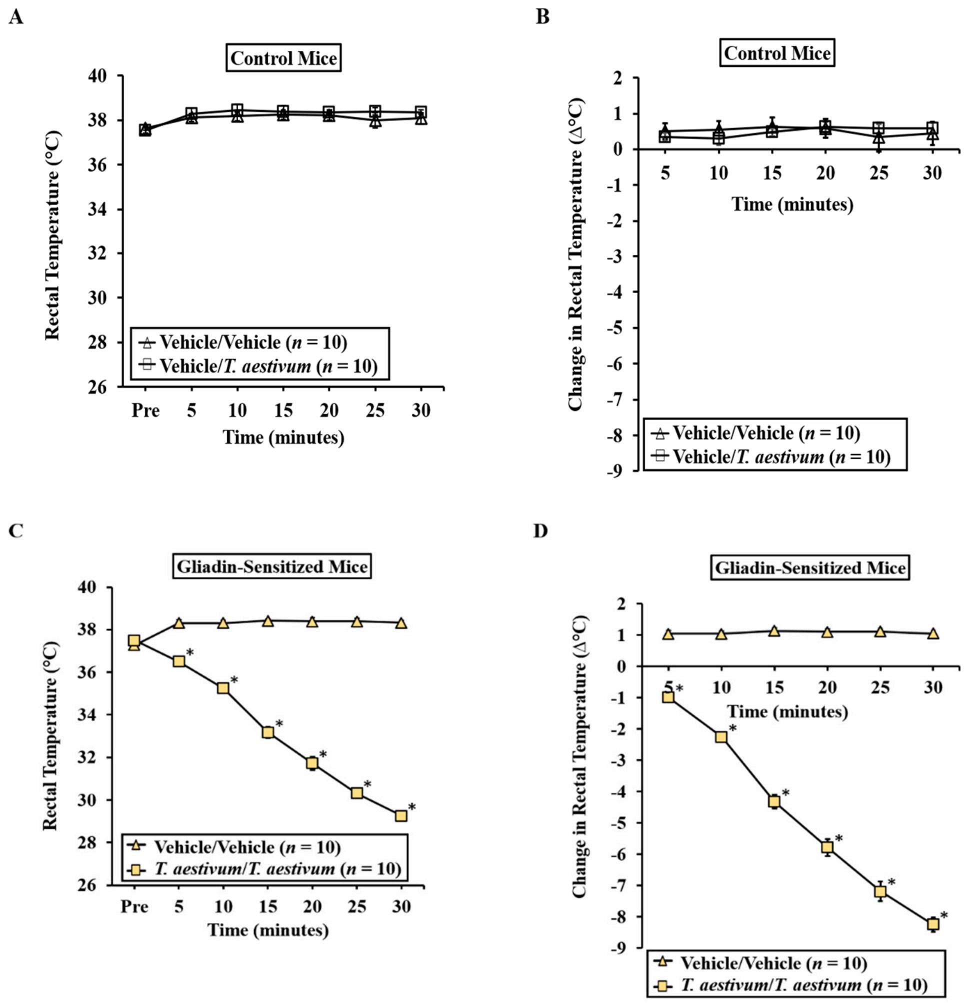

Figure S5. Transdermal sensitization with alcohol-soluble gluten extract from *T. aestivum* is sufficient for eliciting systemic anaphylaxis in Balb/c mice.

Mice were sensitized and systemically challenged with alcohol-soluble gluten extract from *T. aestivum* or vehicle, as described in Materials and Methods. n = 10/group. (A) Actual rectal temperature at indicated time points in control mice challenged with alcohol-soluble gluten extract from *T. aestivum* or vehicle. (B) Change in rectal temperature at indicated time points in control mice challenged with alcohol-soluble gluten extract from *T. aestivum* or vehicle. (C) Actual rectal temperature at indicated time points in alcohol-soluble gluten extract from *T. aestivum* sensitized mice challenged with alcohol-soluble gluten extract from *T. aestivum* or vehicle. (D) Change in rectal temperature at indicated time points in alcohol-soluble gluten extract from *T. aestivum* sensitized mice challenged with alcohol-soluble gluten extract from *T. aestivum* or vehicle. *$p < 0.05$, ANOVA; *n*: number of mice.

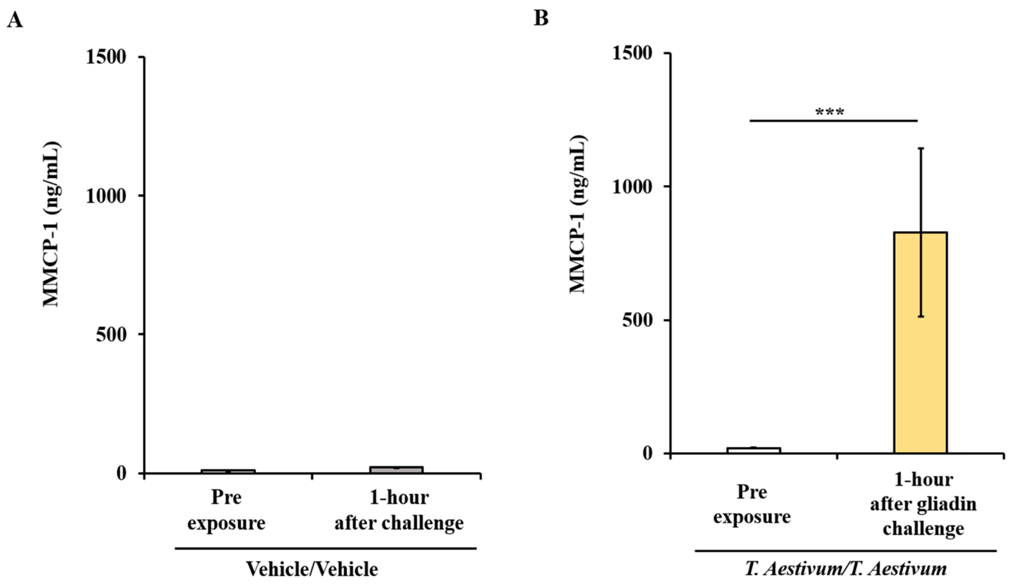

Figure S6. Systemic challenge with alcohol-soluble gluten extract from *T. aestivum* elicits a robust mucosal mast cell response (MMCR) in Balb/c mice.

Mice were sensitized and systemically challenged with alcohol-soluble gluten extract from *T. aestivum* or vehicle, as described in Materials and Methods. Plasma levels of mucosal mast cell protease (MMCP)-1 (ng/mL) in pre and one-hour after challenge were measured via ELISA. n = 10/group. (A) MMCP-1 levels in control mice challenged with vehicle. (B) MMCP-1 levels in allergic mice were challenged with alcohol-soluble gluten extract from *T. aestivum*. ***$p < 0.001$, student's t-test; *n* = number of mice.

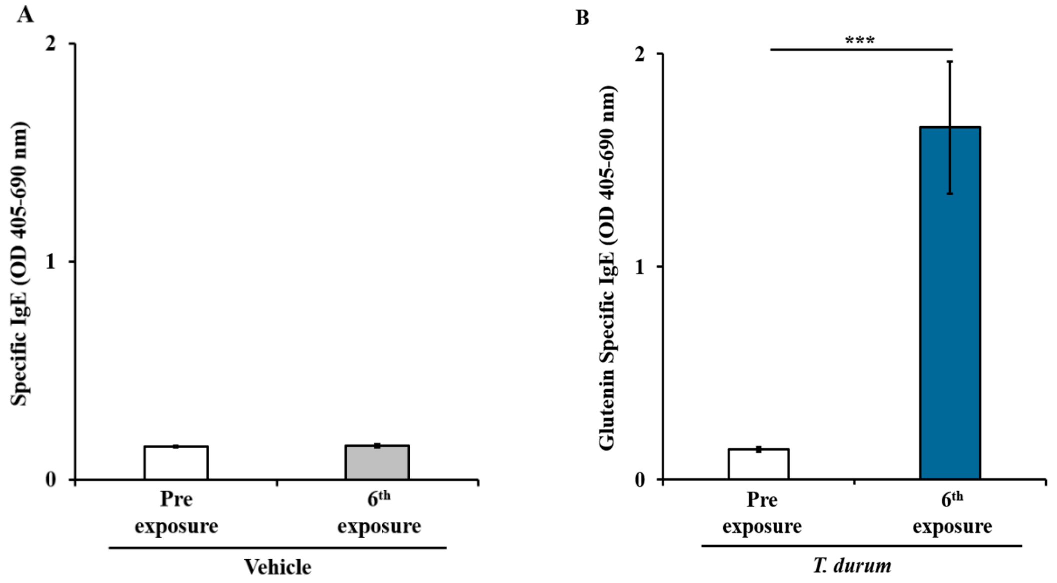

Figure S7. Transdermal exposure of Balb/c mice to acid-soluble gluten extract from *T. durum* (genome AABB) elicited robust specific (s) IgE antibody responses.

Mice were exposed to acid-soluble gluten extract from *T. durum* or vehicle as described in Methods. Plasma collected before the 1st exposure (Pre) and after the 6th exposure (6R) was used in the measurement of sIgE levels (OD 405–690 nm), with a sample size of n = 10/group. (A) sIgE levels in control mice. (B) sIgE levels in sensitized mice. *** $p < 0.001$, student's t-test. Ab: antibody; *n*: number of mice; OD: optical density.

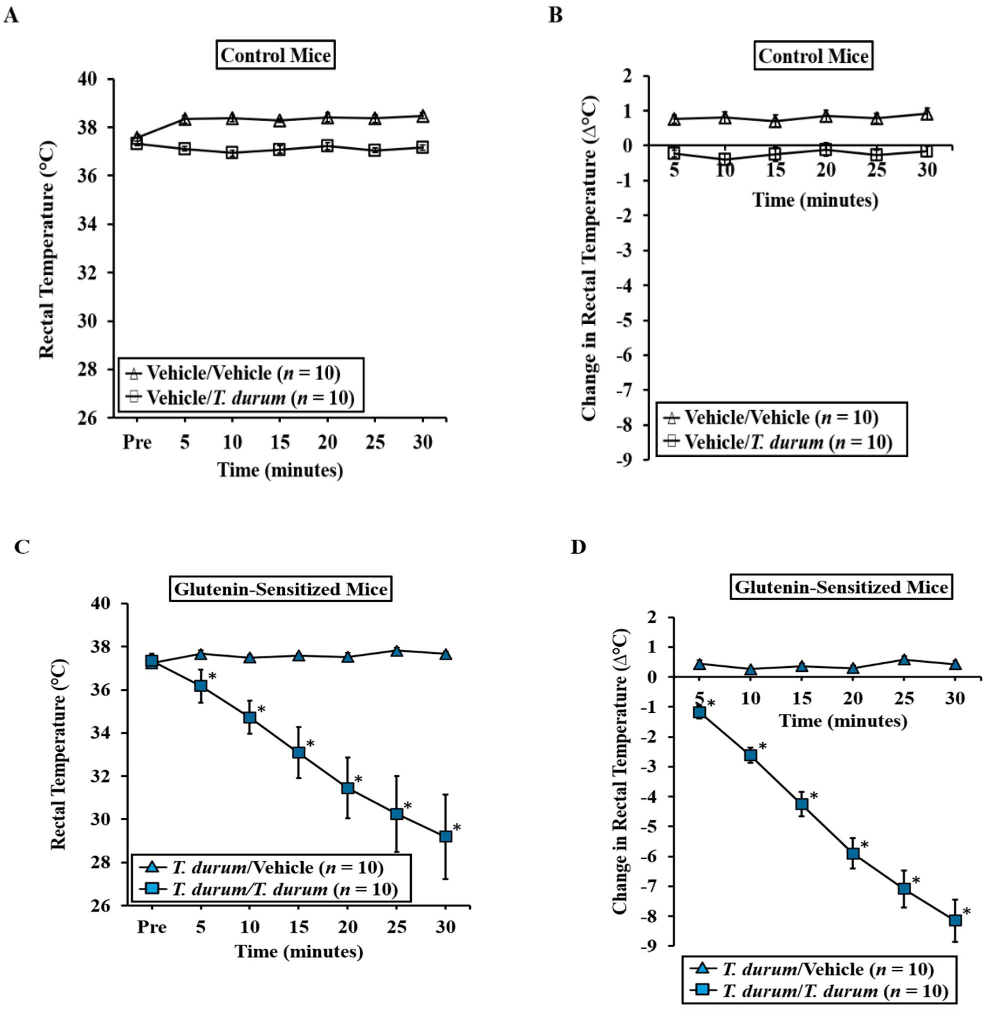

Figure S8. Transdermal sensitization with acid-soluble gluten extract from *T. durum* is sufficient for eliciting systemic anaphylaxis in Balb/c mice.

Mice were sensitized and systemically challenged with acid-soluble gluten extract from *T. durum* or vehicle, as described in Materials and Methods. n = 10/group. (A) Actual rectal temperature at indicated time points in control mice challenged with acid-soluble gluten extract from *T. durum* or vehicle. (B) Change in rectal temperature at indicated time points in control mice challenged with acid-soluble gluten extract from *T. durum* or vehicle. (C) Actual rectal temperature at indicated time points in acid-soluble gluten extract from *T. durum* sensitized mice challenged with acid-soluble gluten extract from *T. durum* or vehicle. (D) Change in rectal temperature at indicated time points in acid-soluble *T. durum* protein extract sensitized mice challenged with acid-soluble *T. durum* protein extract or vehicle. *$p < 0.05$, ANOVA; *n*: number of mice.

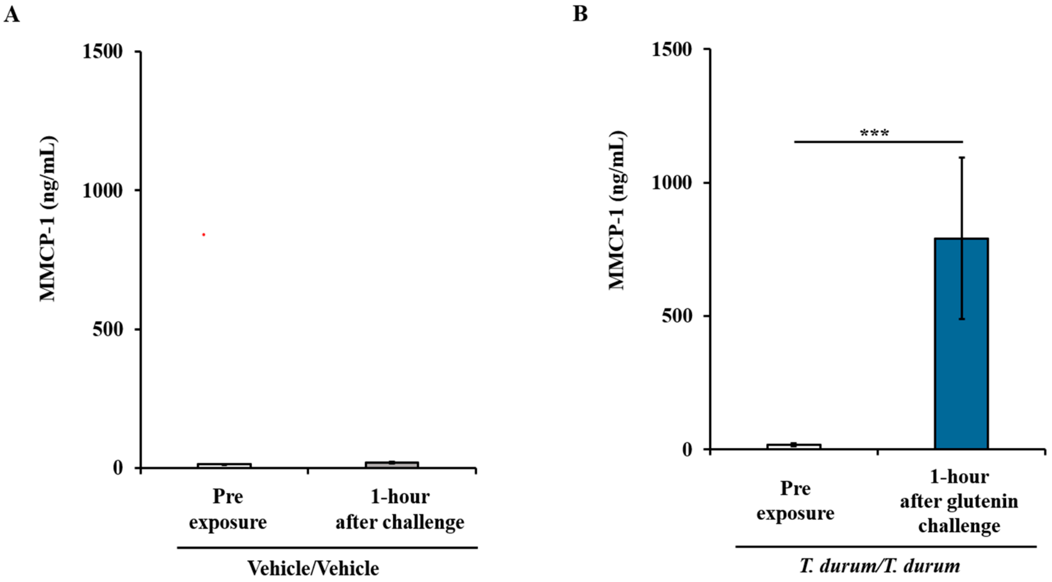

Figure S9. Systemic challenge with *T. durum* acid-soluble gluten extract elicits a robust mucosal mast cell response (MMCR) in Balb/c mice.

Mice were sensitized and systemically challenged with acid-soluble *T. durum* protein extract or vehicle, as described in Materials and Methods. Plasma levels of mucosal mast cell protease (MMCP)-1 (ng/mL) in pre and one-hour after challenge were measured via ELISA. n = 10/group. (A). MMCP-1 levels in control mice challenged with vehicle. (B) MMCP-1 levels in allergic mice challenged with acid-soluble *T. durum* protein extract . ***p < 0.05, student's t-test; *n*: number of mice.

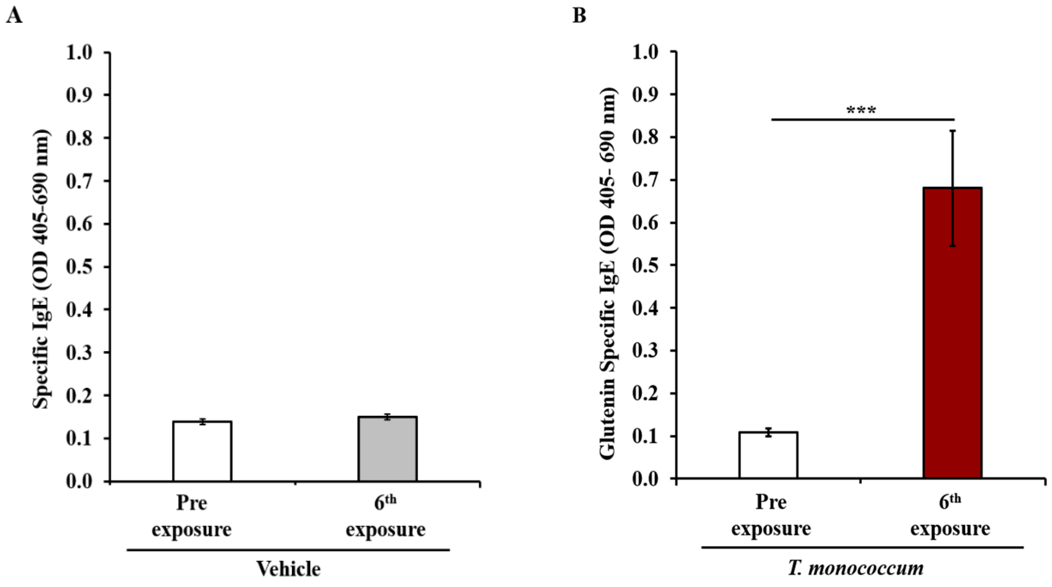

Figure S10. Transdermal exposure of Balb/c mice to acid-soluble gluten extract from *T. monococcum* (genome AA) elicited robust specific (s) IgE antibody responses.

Mice were exposed to acid-soluble gluten extract from *T. monococcum,* or vehicle as described in Methods. Plasma collected before the 1st exposure (Pre) and after the 6th exposure (6R) was used in the measurement of sIgE levels (OD 405–690 nm). n = 10/group. (A) sIgE levels in control mice. (B) sIgE levels in sensitized mice. *** $p < 0.001$, student's t-test. Ab: antibody; *n*: number of mice; OD: optical density.

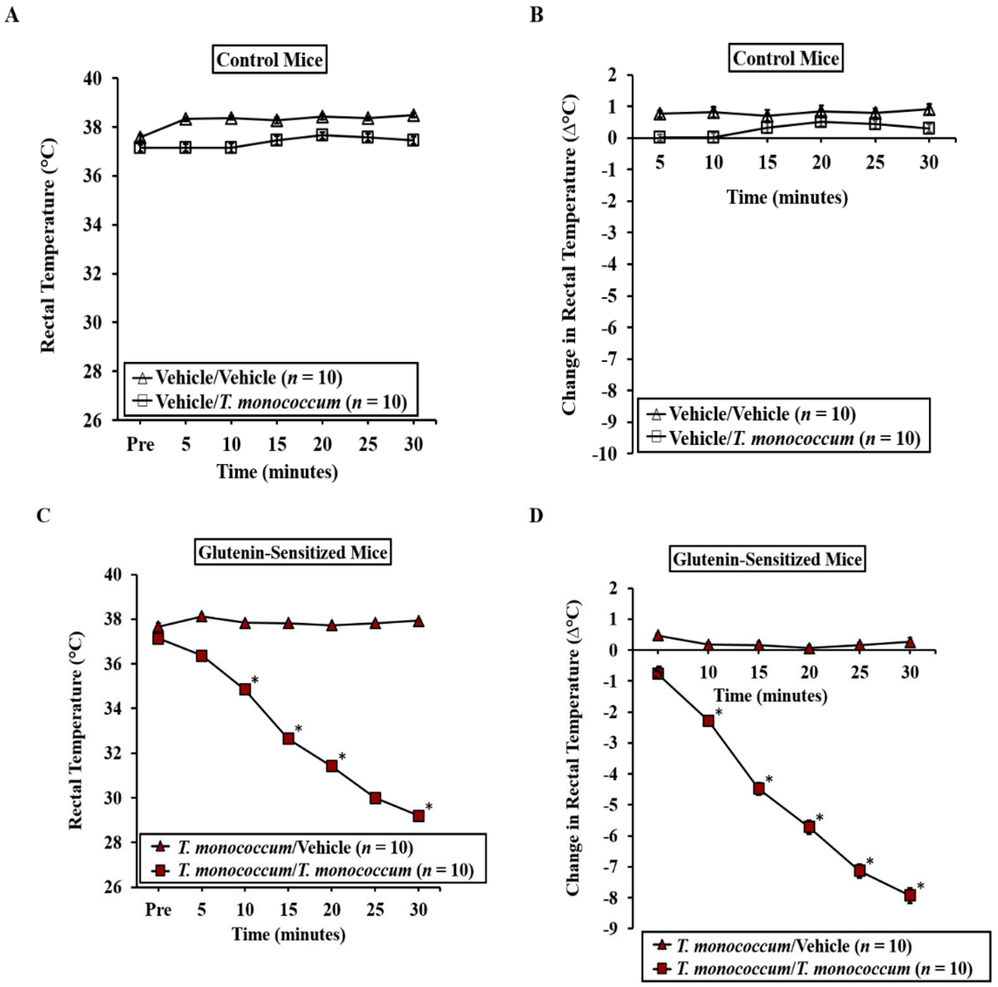

Figure S11. Transdermal sensitization with acid-soluble gluten extract from *T. monococcum* is sufficient for eliciting systemic anaphylaxis in Balb/c mice.

Mice were sensitized and systemically challenged acid-soluble gluten extract from *T. monococcum* or vehicle, as described in Materials and Methods. n = 10/group. (A) Actual rectal temperature at indicated time points in control mice challenged with acid-soluble gluten extract from *T. monococcum* or vehicle. (B) Change in rectal temperature at indicated time points in control mice challenged acid-soluble gluten extract from *T. monococcum* or vehicle. (C) Actual rectal temperature at indicated time points in acid-soluble gluten extract from *T. monococcum* sensitized mice challenged with acid-soluble gluten extract from *T. monococcum* or vehicle. (D) Change in rectal temperature at indicated time points in acid-soluble gluten extract from *T. monococcum* sensitized mice challenged with acid-soluble gluten extract from *T. monococcum* or vehicle. *$p < 0.05$, ANOVA; *n*: number of mice.

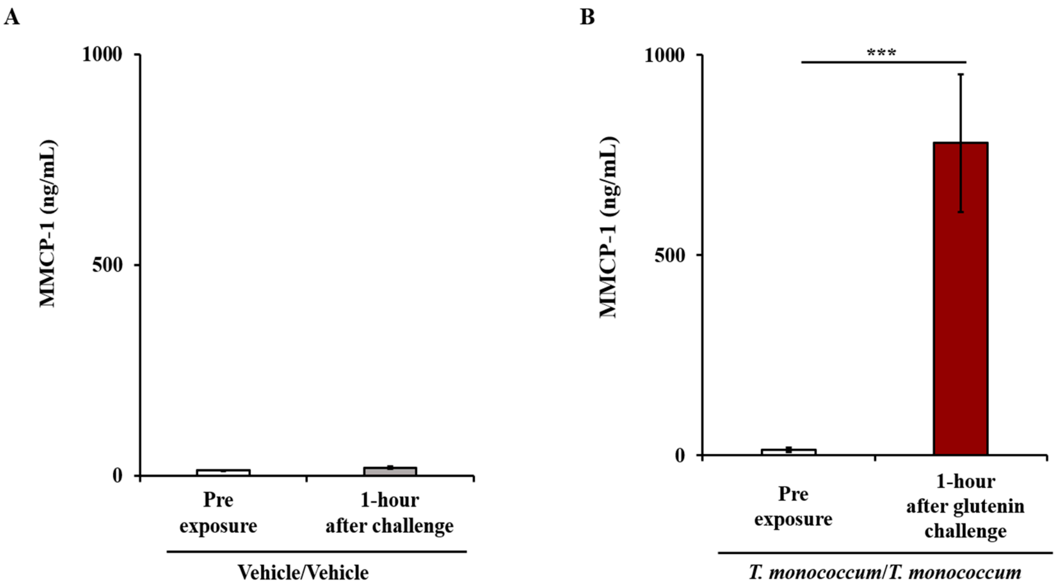

Figure S12. Systemic challenge with acid-soluble gluten extract from *T. monococcum* elicits a robust mucosal mast cell response (MMCR) in Balb/c mice.

Mice were sensitized and systemically challenged with acid-soluble gluten extract from *T. monococcum* or vehicle, as described in Materials and Methods. Plasma levels of mucosal mast cell protease (MMCP)-1 (ng/mL) in pre and one-hour after challenge were measured via ELISA. n = 10/group. (A). MMCP-1 levels in control mice challenged with vehicle. (B) MMCP-1 levels in allergic mice challenged with acid-soluble gluten extract from *T. monococcum*. ***$p < 0.05$, student's t-test; *n*: number of mice.

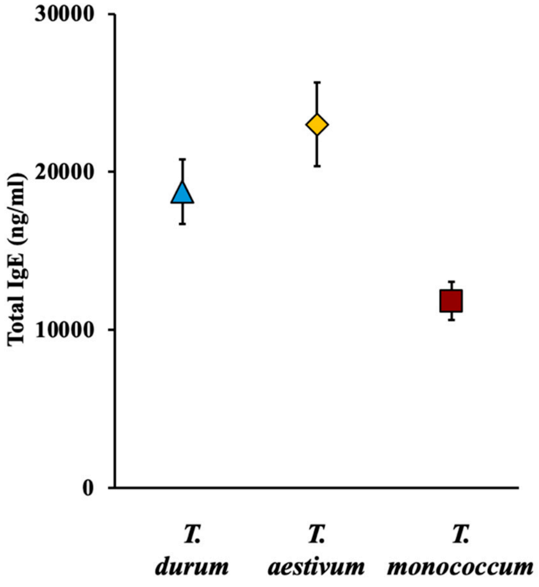

Figure S13. A comparative map of glutenin allergenicity using total IgE measurements in this mouse model.

Mice were sensitized with acid-soluble gluten (glutenin) extract from three wheat species as indicated using the protocol in Materials and Methods. Plasma levels of total IgE in pre and after 6th skin exposure were measured using the ELISA method. After subtracting the pre levels from the post skin exposure, data was used to create the comparative map of glutenin allergenicity as shown in the figure; *T. durum* vs. *T. monococcum* and *T. aestivum* vs. *T. monococcum*: *$p < 0.05$, Student's t test; n = 10/group.
